# Prevalence, risk factors and awareness of albuminuria on a Canadian First Nation: A community-based screening study

**DOI:** 10.1186/1471-2458-12-290

**Published:** 2012-04-20

**Authors:** James Michael Zacharias, T Kue Young, Natalie D Riediger, Joanne Roulette, Sharon G Bruce

**Affiliations:** 1 Section of Nephrology, Department of Internal Medicine, Health Sciences Centre, University of Manitoba, Room GE644, 820 Sherbrook St, Winnipeg, Manitoba, R3A 1R9, Canada; 2 Dalla Lana School of Public Health, University of Toronto, 155 College St, Toronto, Ontario, M5T 3M7, Canada; 3 Department of Community Health Sciences, University of Manitoba, S113-750 Bannatyne Ave, Winnipeg, Manitoba, R3E 0W3, Canada; 4 Sandy Bay Ojibway First Nation, Sandy Bay Health Centre, Box 110, Marius, Manitoba, R0H 0T0, Canada

**Keywords:** Canada, First Nation, Albuminuria, End-stage renal disease, Kidney, Diabetes

## Abstract

**Background:**

Both diabetic and non-diabetic end stage renal disease (ESRD) are more common among Canadian First Nations people than among the general Canadian population. The purpose of this research was to determine the prevalence of and risk factors for albuminuria in a Canadian First Nation population at high risk for ESRD and dialysis.

**Methods:**

Data from a community-based screening study of 483 residents of a Plains Ojibway First Nation in Manitoba was used. Participants provided random urine samples. Proteinuria was defined as any dipstick positive for protein (≥1 g/L) or those with ACR in the macroalbuminuric range (≥30 mg/mmol) on at least one sample. Microalbuminuria was defined as ACR ≥2 mg/mmol for males and ≥2.8 mg/mmol for females. Other measures included fasting glucose, haemoglobin A_1c_, triglycerides, cholesterol, blood pressure, height, weight and waist and hip circumferences.

**Results:**

Twenty percent of study participants had albuminuria, (5% proteinuria and 15% microalbuminuria). Of participants with diabetes, 42% (56/132) had albuminuria compared to 26% (7/27) among those with impaired fasting glucose and 10% (30/303) among those with normal glucose tolerance. Only 5.3% of those with albuminuria were aware of any degree of renal disease. In a multivariate logistic regression, independent associations with albuminuria were male gender [p = 0.002], increasing fasting glucose [p <0.0001], years diagnosed with diabetes [p = 0.03], increasing systolic blood pressure [p = 0.009], and increasing body mass index (BMI) [p = 0.04].

**Conclusions:**

The independent association between BMI and albuminuria has not been previously reported among indigenous populations. There is a high prevalence of albuminuria in this Canadian First Nation population; the high proportion of patients with diabetes and undiagnosed kidney disease demonstrates the need for screening, education and intervention to halt the progression and development of albuminuria and ultimately ESRD and CVD.

## Background

The incidence of end-stage renal disease (ESRD) among Canadian First Nations people is 2.5-4 times greater than among the non-First Nations population [1-3] and the most common cause is diabetic nephropathy. The earliest indicator of diabetic renal disease is microalbuminuria [4-10], which appears 5-10 years prior to the onset of overt proteinuria. Proteinuria is a sign of more advanced renal disease and is a precursor to renal failure [11]. Importantly, albuminuria is a strong and independent predictor of cardiovascular and all cause mortality [12]. Additionally, the impact of renal disease on health care costs and quality of life for patients and their caregivers is substantial [13-16]. Blood pressure and glucose control among those with diabetes and/or chronic kidney disease, with either medication and/or lifestyle changes have shown to be effective in preventing progression to advanced renal disease [17-19]. Therefore screening for albuminuria in high risk populations may be one component of an effective community-based renal disease prevention strategy.

While the excess burden of diabetes among First Nations populations compared to the general Canadian population is well established [20], research on diabetes-related kidney disease among Canadian First Nations populations is more limited. A retrospective population-based study from Saskatchewan for the years 1981-90 revealed that among those with diabetes, the rate of ESRD in First Nation people was 7 times greater than among non-First Nation people [2]. The prevalence of microalbuminuria among a sample of 601 First Nation residents in northern Saskatchewan was 24% among those with diabetes, and 9% among those without [21]. Non-diabetic diseases, such as glomerulonephritis, that can lead to ESRD and which can present with albuminuria and hematuria are also significantly increased in First Nation people [3]. According to the Canadian Diabetes Association Clinical Practice Guidelines, First Nations adults with at least one risk factor should be screened for diabetes every 1-2 years and those with type 2 diabetes should be screened for albuminuria at diagnosis and annually thereafter [22]. Despite these recommendations and the increased burden of diabetic and non-diabetic renal disease, few population-based screening studies for albuminuria among Canadian First Nation populations have been performed. The study community reported in this article has a high prevalence of ESRD (5%), diabetes (29%) and impaired fasting glucose (7%) [23]. Thus, the community is at high risk for diabetic renal complications. Therefore the purpose of this paper is to determine the prevalence and determinants of albuminuria in a Canadian First Nation population, and to begin planning for secondary prevention strategies.

## Methods

The data for this paper are from a previously described larger screening study for diabetes and diabetes complications [23] conducted in 2003 among adult members of the Sandy Bay First Nation, located about 200 km northwest of Winnipeg, Manitoba, Canada, with year round road access. Its population in December 2001 was 2,968, of which 52% were under the age of 18. Inclusion criteria were: non-pregnant, community member, and 18 years and older. A total of 483 community members participated; 36% of all eligible adults (n = 1356). That is, there were 1356 eligible adults in the community, according to the inclusion criteria, and 483 chose to participate. Estimates of albuminuria were obtained on the 468 participants who had completed all components of the study, which included anthropometrics (height, weight, and waist and hip circumferences); a demographic and health status questionnaire; fasting blood samples for glucose, A1c,and lipids; and random urine sample. Although albuminuria is not the best clinical tool to identify kidney disease, for an epidemiological screening study, albuminuria is an excellent predictor for ESRD, CVD, and all-cause mortality [12]. This study was approved by the Health Research Ethics Board at the University of Manitoba.

### Measures

Urine albumin:creatinine ratio (ACR) was determined using the Bayer® DCA 2000™ Point-of-Care Analyzer, which is highly accurate and reproducible [24,25]. Each urine sample was first tested by a Multistix Reagent strips for presence of protein, and blood. Participants’ whose samples tested “trace or more” for blood or ≥ 1 g/L protein were requested to return on another day. Samples negative for blood and protein were loaded into the DCA 2000™. Microalbuminuria was defined as no positive dipstick reading for protein and at least one test with ACR >2.8 mg/mmol for women and >2.0 mg/mmol for men. Individuals with dipstick positive proteinuria (>1 g/L) or those with ACR in the macroalbuminuria of proteinuria range (≥30 mg/mmol) on at least one sample were considered to have proteinuria. A maximum of three samples were obtained.

Venous samples for glucose, were drawn after an overnight 12-hour fast. Participants with fasting glucose values ≥5.8 mmol/L were requested to return for a second sample; the average of the two samples was used in analysis. Diabetes was defined as a fasting blood glucose ≥7.0 mmol/L. Impaired fasting glucose was defined as fasting blood glucose of 6.1-6.9 mmol/L, as per the Canadian Diabetes Association [26]. Samples were analyzed at the Clinical Chemistry Lab at the Health Sciences Centre, Winnipeg, Manitoba.

Blood pressure was measured on site by a registered nurse or a trained research assistant, working under the supervision of the registered nurse. The average of two readings was used. Hypertension was defined as a blood pressure >140/90 mm Hg for those without diabetes, ≥130/80 mm Hg for those with diabetes and/or albuminuria, according to the American Diabetes Association [27,28], or a previous diagnosis treated with medication. Anthropometric measures including height, weight, and waist and hip circumferences were completed using standard techniques [29]. Current and past smoking status and number of cigarettes smoked per day were determined using a standardized questionnaire. Pack years was calculated as number of packs per day (1 pack = 20 cigarettes) multiplied by number of years smoked. Standard demographic information and diabetes history were derived via questionnaire. Awareness of disease states by participant, such as diabetes, hypertension and kidney disease, was asked by a simple direct question in similar fashion to the NHANES III study cohort [30].

### Statistical analyses

Data are presented as mean (standard deviation) for continuous variables, median (range) for continuous variables that do not follow a normal distribution, and as n (percent) for categorical variables. Demographic, anthropometric and health characteristics were compared using *t*-tests for normally distributed continuous variables, χ^2^ tests for categorical variables and Mann–Whitney U non-parametric tests for non-normally distributed continuous variables. Univariate and multiple logistic regression (backwards stepwise) were used to determine associations with albuminuria. Continuous variables were also explored as categorical variables to determine appropriateness of assumptions of linearity. Those variables that were found to be significant in univariate analysis, as well as independent of age, were included as potential variables in the backwards stepwise logistic regression model. Tests were two-tailed with p < 0.05 considered significant. Statistical analyses were performed using SPSS (version 16).

## Results

The study sample was representative of the larger community for age, sex, education and employment status [23]. That is, the sample had similar proportions of the previously listed characteristics as the study population. Nine subjects were ineligible due to incomplete data and 6 were excluded from the albuminuria analysis due to persistent hematuria, leaving 468 subjects for albuminuria analysis. Participants with persistent hematuria were excluded due to the inability to obtain an ACR and we could not determine the cause of the hematuria. These individuals were referred for clinical evaluation. However, most individuals whose first test was positive for hematuria were found not to be hematuric on subsequent tests and were included in analysis. The majority of these participants were women who were tested close to their menstrual period.

Demographic and health risk characteristics of the study population are described in Table 1. Both sexes were equally represented. The population was young and the mean age was similar for men and women (37 for men; 38 for women). Less than half the study sample had completed grade 9. The study population had high rates of cardiovascular risk factors; three-quarters of participants were current smokers, 43% had hypertension, 35% had diabetes or prediabetes and obesity was highly prevalent.

**Table 1:** Demographic and health risk characteristics of the study sample

Characteristic	Value^1^
Age (years)	38 (12)^a^
Female	253 (52)
Grade 9 or higher	220 (47)
Employed	137 (29)
Ever smoked	391 (82)
Current smoker	349 (74)
Hypertension^2^	201 (43)
Body Mass Index (kg/m^2^)	31.5 (7)^a^
Diabetes^3^	140 (29)
Impaired fasting glucose^4^	30 (6.2)
Average fasting blood glucose (mmol/L)	5.4 (3.4, 20.6)^b^
Albuminuria^5^	95 (20)
Aware^6^ of diabetes	107(76)
Aware of hypertension	127(63)
Aware of kidney disease	5(5)

^1^Data are reported as n (percentage), mean (standard deviation)^a^ or median (range)^b^.

^2^Hypertension is defined as a blood pressure >140/90 mm Hg for those without diabetes, ≥130/80 mm Hg for those with diabetes, or a previous diagnosis with medications.

^3^Diabetes is defined as fasting glucose ≥7.0 mmol/L or previous diagnosis.

^4^Impaired fasting glucose is fasting glucose between 6.1 mmol/L and 6.9 mmol/L with no previous diagnosis of diabetes.

^5^Albuminuria is albumin:creatinine ratio >2.8 mg/mmol for women and >2.0 mg/mmol for men.

^6^Awareness of disease states refers to participant awareness.

Twenty-five percent of the sample returned for at least a second random urine test (Table 2). However, of those who had abnormal samples (ie. hematuria or proteinuria) on their first test, 79% returned for a second or third test. Albuminuria was present in 95/468 or 20% of participants. Twenty-five participants (5%) had proteinuria and 70 (15%) had microalbuminuria. In univariate analysis [OR (95% CI) p-value], age [1.039 (1.008, 1.072) p = 0.014], diabetes [11.474 (4.209, 31.279) p < 0.001], systolic blood pressure [1.039 (1.019, 1.061) p < 0.001], diastolic blood pressure [1.051 (1.018, 1.086) p = 0.002], fasting glucose [1.257 (1.155, 1.367) p < 0.001] and hypertension [4.390 (1.709, 11.277) p = 0.002] were associated with proteinuria. There was a non-significant trend for lower odds of proteinuria among females [0.428 (0.181, 1.012) p = 0.053]. BMI, ever smoker, and years diagnosed with diabetes were not associated with proteinuria. For all other analysis, those with proteinuria and microalbuminuria were combined for analysis due to small numbers and equivalence on the following characteristics: age, sex, smoking status and mean years smoked. However, the proportion of those with diabetes was greater among the proteinuria group compared to the microalbuminuria group. Eighty percent (20/25) of those with proteinuria had diabetes.

**Table 2:** Albuminuria category by number of tests done

Number of urine tests done	Albuminuria Category			
	Normo-albuminuria	Microalbuminuria^a^	Proteinuria^b^	Totals
1	321 (69%)	22 (4.7%)	6 (1.3%)	349 (75%)
2	48 (10%)	39 (8.2%)	14 (3%)	101 (22%)
3	4 (0.8%)	9 (1.9%)	5 (1.1%)	18 (3.8%)
Totals	373 (80%)	70 (15%)	25 (5.3%)	468

^a^Microalbuminuria was defined as no positive dipstick reading for protein and at least one test with ACR >2.8 mg/mmol for women and >2.0 mg/mmol for men.

^b^Individuals with dipstick positive proteinuria (>1 g/L) or those with ACR in the macroalbuminuria of proteinuria range (≥30 mg/mmol) on at least one sample were considered to have proteinuria.

**Figure 1:**
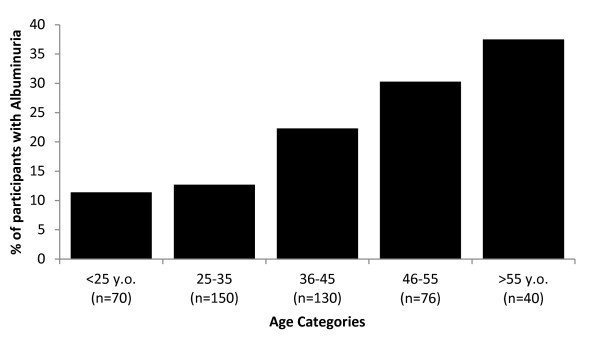
**Percent albuminuria by age categories.** There is a significant linear association between age group and proportion of those with albuminuria (p < 0.001). ‘n’ refers to the total number of participants in respective age category.

Participants with and without albuminuria are compared in Table 3. Those with albuminuria were older (p < 0.001) and albuminuria prevalence progressively increased with age (Figure 1). Men were more likely to have albuminuria than women (p = 0.01). Interestingly, those with albuminuria were less likely to report ever having smoked (p = 0.007). However, neither years of smoking nor pack-years smoked were significantly different between groups. Participants with albuminuria were more likely to be hypertensive by any measure. Seventy percent (70%) of those with albuminuria had hypertension compared with 36% of those without albuminuria (p < 0.0001). In this regard, mean blood pressures were significantly higher in the group with albuminuria compared to those without. Target blood pressure in those with albuminuria is generally recommended to be <130/80 mm Hg, however, this was achieved in only 34% of those with albuminuria.

**Table 3:** Comparison of those with and without albuminuria

Characteristic	Albuminuria (n = 95)	No albuminuria (n = 373)	P
Age (years)	42 (13)	36 (12)	<0.001
Sex			
Female	38 (40.4)	201 (54.0)	0.02
Male	56 (59.6)	171 (46.0)	
Ever a smoker	67 (72.0)	311 (84.1)	0.007
Years smoked^a^	12 (0,50)	10 (0,50)	0.941
Pack years smoked^a^	1.5 (0,88)	2.6 (0,74)	0.291
Previous hypertension diagnosis	38 (42.7)	78 (21.9)	<0.001
Hypertension^1^	64 (68.8)	131 (35.7)	<0.001
Systolic BP (mm Hg)^b^	134 (19)	125 (15)	<0.001
Diastolic BP (mm Hg)^b^	80 (12)	76 (10)	<0.001
Blood pressure ≥130/ 80 mm Hg^2^	62 (66.0)	189 (51.2)	0.01
Body Mass Index (kg/m^2^)^b^	32.9 (5.79)	31.1 (7.25)	0.03
Diabetes^3^	57 (60.6)	77 (20.7)	<0.001
Dysglycemia^4^	64 (68.1)	97 (26.1)	<0.001
Fasting glucose (mmol/L)^b^	9.7 (4.6)	6.2 (2.6)	<0.001
Hematuria^5^	8.4	2.9	0.001

Data are reported as n (percentage), mean (standard deviation)^a^, median (range)^b^.

^1^Hypertension is defined as a previous diagnosis of hypertension or blood pressure ≥140/90 mm Hg.

^2^Blood pressure ≥130/80 mm Hg includes only participants with previous diagnosis of diabetes or hypertension.

^3^Diabetes is defined as on oral hypoglycemics, self declared diabetes or has a fasting glucose ≥7.0 mmol/L.

^4^Dysglycemia is defined as previous diagnosis of diabetes or a fasting plasma glucose ≥6.1 mmol/L.

^5^Hematuria was a positive dipstick for hemoglobin.

**Figure 2:**
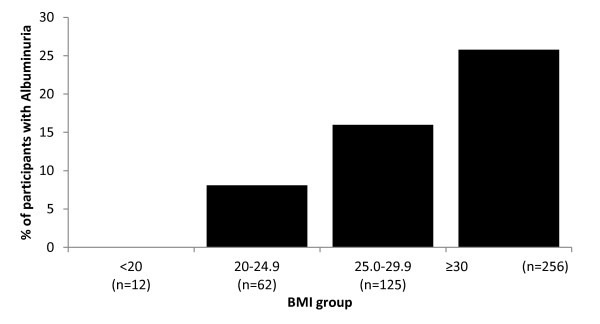
**Percent albuminuria by BMI categories.** There is a significant linear association between BMI category and proportion of those with albuminuria (p < 0.001). ‘n’ refers to the total number of participants in respective age category.

Participants with albuminuria were significantly heavier and had a higher BMI (Figure 2). This increased weight and BMI may be related to the greater proportion of participants in the albuminuric group with diabetes and impaired fasting glucose. Fasting glucose was also higher among those with albuminuria compared to those without (p < 0.001). While almost 60% of those with any degree of albuminuria had diabetes, 42% of those with diabetes were albuminuric.

Odds ratios and 95% CI are listed in Table 4 for both univariate and multivariate logistic regression. Backwards stepwise multivariate logistic regression was performed. Univariate analysis indicated the odds of having microalbuminuria increased 4% for each year increase in age. However, age was not significant in the final multivariate model. When both fasting glucose and years of diabetes were removed from the model and age was included, age became significant suggesting high multicolinearity between the diabetes and age (model not shown). Females were less likely to have microalbuminuria than males, which remained significant in the multivariate model. Smokers were less likely to have albuminuria than non-smokers and while smoking appeared protective in univariate analysis, it did not achieve significance in the multivariate model.

**Table 4:** Univariate and multivariate logistic regression with albuminuria as an outcome

	Odds ratio (95%CI)	p-value
**Univariate logistic regression**		
Age, years	1.040 (1.021,1.059)	<0.0001
Sex		
Male	1	
Female	0.560 (0.354, 0.885)	0.01
Ever smoked		
No	1	
Yes	0.485 (0.285,0.825)	0.01
Diabetes mellitus		
No	1	
Yes	7.718 (4.412, 13.5)	<0.001
Average fasting glucose, mmol/L	1.27 (1.192, 1.352)	<0.001
Years diagnosed with DM	1.127 (1.078, 1.179)	<0.001
Hypertension		
No	1	
Yes	2.860 (1.790, 4.560)	<0.001
Systolic blood pressure, mmHg	1.032 (1.019, 1.046)	<0.001
Diastolic blood pressure, mmHg	1.034 (1.013, 1.055)	<0.005
BMI	1.036 (1.004, 1.070)	0.03
Hematuria		
No	1	
Yes	3.079 (1.201, 7.877)	0.02
**Multivariate logistic regression**		
Sex		
Male	1	
Female	0.407 (0.230, 0.771)	0.002
Average fasting glucose, mmol/L	1.200 (1.115, 1.29)	<0.001
Years diagnosed with DM	1.057 (1.004, 1.113)	0.03
Systolic blood pressure, mmHg	1.020 (1.005, 1.035)	0.01
BMI	1.042 (1.001, 1.085)	0.04

DM, diabetes mellitus; BMI, body mass index.

Those with diabetes were seven times more likely to have albuminuria than those without diabetes. In addition, for each mmol/L increase in average fasting glucose, albuminuria increased by 27% in the univariate model, and 20% when controlling for sex, years diagnosed with diabetes, systolic blood pressure and BMI. Length of time with diabetes was also a significant predictor of albuminuria and confirms the natural history of diabetic nephropathy. Systolic blood pressure was independently associated with albuminuria in the multivariate model and risk of albuminuria increased by 2% for every mm Hg increase in systolic blood pressure. Lastly, weight and BMI were both significant alone, but BMI was an independent predictor of albuminuria in the multivariate analysis, after accounting for average fasting glucose, gender, years diagnosed with diabetes mellitus and systolic blood pressure [1.042 (1.001, 1.085), p = 0.04].

In contrast to participants with diabetes and hypertension, very few with albuminuria were aware of their condition. Participant awareness of kidney disease increased with severity such that 17% of those with proteinuria were aware of kidney disease while awareness kidney disease in those with microalbuminuria was only 1% (Table 1).

## Discussion

High prevalence of diabetes and renal complications of diabetes (ESRD) have been previously described among Canadian First Nations people [1,2,31-33]. Significant rates of renal complications (ESRD) among non-diabetic First Nations people are also seen [3,34]. Microalbuminuria is well recognized as a significant precursor to either diabetic or non-diabetic renal disease, and has been described in both those with and without diabetes. In the current population-based study, albuminuria rates of 20% among the total population were as high as among other high-risk populations such as the Pima in the southwestern United States [4,7,35]. Among those with diabetes in our study, 42% had albuminuria, which is substantially greater than the prevalence among the general US population (28.8%) as described in the NHANES III study [36], and the 24% estimated in a Canadian First Nation population-based study in Saskatchewan [21]. The present study prevalence is comparable to the American Pima and Zuni populations in which 47% and 52% of the diabetic population, respectively, had albuminuria [7,15].

The current study found female gender protective for albuminuria, independent of fasting glucose, years with diabetes, systolic blood pressure or BMI. This association was also found in the Strong Heart Study [37]. This contrasts with the NHANES III and Pima Indian non-diabetic data, in which women had significantly more albuminuria than men [7,36]. The NHANES III study, however, did not use the lower cut-off for ACR in males, as did the current study. A different ACR cut-off due to higher excreted creatinine in males has been supported by many investigators and is supported by the Canadian Diabetes Association Clinical Practice Guidelines [26,38-40], and may account for the differences between the NHANES and Pima Indian non-diabetic data and the current study. Furthermore, in the Pima diabetic population and the Zuni Indian population studies, no sex differences were seen [4,7,35]. Mortality rates among men with albuminuria tend to be higher than in women [41], and therefore the increased albuminuria among men in our study is concerning.

BMI was also independently related to increased prevalence of albuminuria in the study sample. Although increased BMI has been described as a risk factor for albuminuria and proteinuria [42] and high BMI is an issue among those of First Nations ancestry [43-45], to our knowledge, BMI has not been previously described as an independent risk factor for microalbuminuria in the Canadian First Nations population.

While age has been found to be significantly associated with albuminuria [36], age was not included in our final multivariate model, likely due to high multicolinearity with duration of diabetes and diabetes itself. However, the average age of those with albuminuria increased by glycemic status (i.e., non-diabetes, IFG and diabetes). While by no means confirmatory, this suggests a possible difference in lead time. As albuminuria is a risk factor for diabetes [46], those younger individuals with dysglycemia and even without diabetes, may develop diabetes as they age.

Participant awareness of kidney disease in a First Nation community is reported here for the first time. Awareness of kidney disease within the study population was low compared to other disease states such as diabetes and hypertension. Awareness of kidney disease was also lower than that found among the general U.S. population where 24% of participants reported being aware of their kidney disease [29]. Disease awareness is related to many factors at both the individual and systems levels. At the individual level, reported awareness of disease can be limited by patient understanding and denial; that is, although care providers may inform patients of their illness, patients may not integrate that information. Another factor affecting awareness is lack of screening or diagnostic testing on the part of health care providers, thereby preventing detection. At the systems level, access to physicians on the part of patients and suitable practice environments for physicians (i.e., access to and remuneration for diagnostic testing) can either enable or act as barriers to preventive practice. Factors influencing poor disease awareness among our study population could not be determined. Thus, further research is required to address the lower than anticipated awareness in this high-risk population.

The study is subject to limitations. The sample size is limited and was not randomly selected from the eligible population. However, our sample was representative of the eligible study population on demographic factors (i.e., age, sex, employment, education) and, as previously reported [23], was not over-represented by the ill or infirmed. In fact, only 38% of those known to have diabetes at the start of the study were participants. In addition, only three out of 10 individuals with previous amputations participated in the study, and none of the 15 individuals with ESRD participated. Therefore, we are confident that our sample represents the larger community. However, the results may not be generalizable beyond the study population because we are not able to determine if the study population is representative of First Nations communities across Canada. Furthermore, the lack of availability of serum creatinine to further indicate severity of renal dysfunction is also a limitation. Lastly, those participants that had a normal ACR did not complete repeat testing.

## Conclusions

Albuminuria is an important complication of diabetes and a risk factor for ESRD and cardiovascular disease. Identification of relevant risk factors is therefore an important activity that can assist in the development of targeted prevention efforts. Interestingly, we found the presence of significant numbers of people with microalbuminuria and hematuria without other risk factors, confirming the presence of non-diabetic renal disease. Further investigations should be focused on this group; first to help ascertain the cause of their hematuria or albuminuria, and second to intervene appropriately. Lack of awareness of early renal disease among study participants is a concern and efforts to understand the phenomenon and improve awareness will be undertaken in partnership with the community. Ongoing longitudinal follow-up of the individuals in this study would further our understanding of the significance of individual health determinants. In addition, further follow-up would give important information about the natural history of albuminuria in this population. The response to treatment and community intervention strategies could also be assessed.

## Abbreviations

ACE: Angiotensin-converting enzyme; ACR: Albumin-creatinine ratio; ARB: Angiotensin receptor blocker; BMI: Body mass index; ESRD: End-stage renal disease.

## Competing interests

The authors declare that they have no competing interests.

## Authors’ contributions

JZ conducted data analysis, interpretation, and preparation of the manuscript. KY is the principal investigator and was involved in the conception and design of the study, analysis and has reviewed this manuscript for intellectual content. NDR assisted in data analysis and preparation of the manuscript for publication. JR is the Director of the health centre in the community and was involved in the conception and design of the study. SB contributed to the conception and design of the study as well as acquisition of the data, analysis and preparation of the manuscript. All authors read and approved the final manuscript.

## Pre-publication history

The pre-publication history for this paper can be accessed here:

http://www.biomedcentral.com/1471-2458/12/290/prepub
